# First person – Sarah Escuin and Saba Raza-Knight

**DOI:** 10.1242/dmm.050105

**Published:** 2023-03-14

## Abstract

First Person is a series of interviews with the first authors of a selection of papers published in Disease Models & Mechanisms, helping researchers promote themselves alongside their papers. Sarah Escuin and Saba Raza-Knight are co-first authors on ‘ Dual mechanism underlying failure of neural tube closure in the *Zic2* mutant mouse’, published in DMM. Sarah conducted the research described in this article while a research associate in the lab of Prof. Andrew Copp at University College London, London, UK, investigating the mechanisms of neurulation and identifying the causes underlying neural tube defects. Saba conducted the research described in this article while an MB PhD student in the lab of Prof. Andrew Copp and Prof. Nicholas Greene at University College London, London, UK. She is now a specialty trainee in neurosurgery at Salford Royal Hospital, Salford, UK, and an honorary clinical lecturer at the University of Manchester, Manchester, UK, investigating neurovascular disorders, cancer biology and genetics, and hydrocephalus, with an active role in medical education.


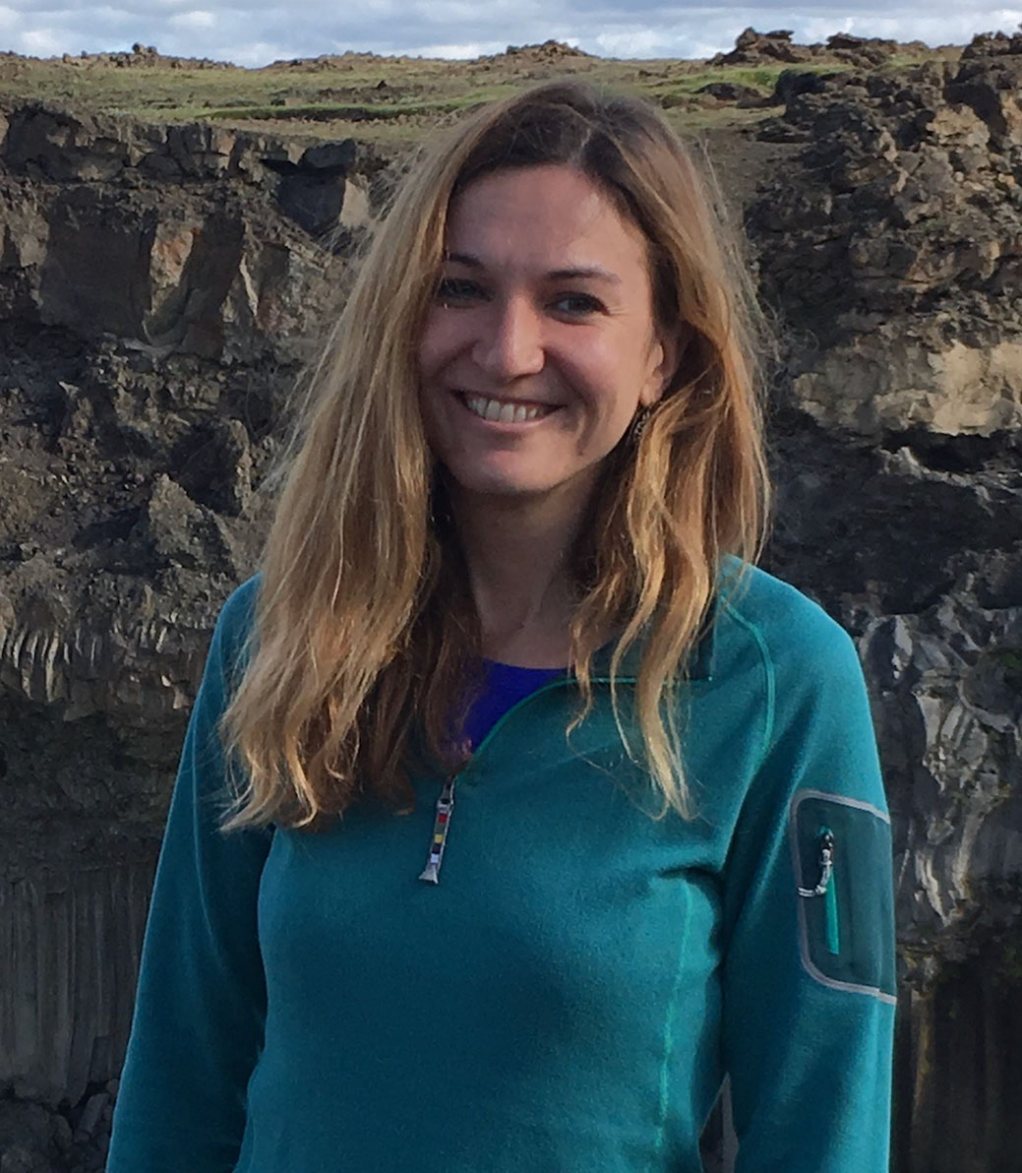


**Sarah Escuin**

**How would you explain the main findings of your paper to non-scientific family and friends?**

**S.E./S.R.-K.:** Open spina bifida is a birth defect found in approximately six in 10,000 live births. It occurs when the lower spine does not form properly. In embryos, the spine forms when a flat sheet of neural tissue rolls up to form a tube. It closes from top to bottom with a ‘zippering’ mechanism. If the rolling up or zippering fail, the developing spinal cord lies open and is damaged *in utero*. Babies with spina bifida have varying degrees of leg weakness, bowel and bladder problems, infection and hydrocephalus (a build-up of fluid in the brain); these can cause death or lifelong disability.

We studied a mouse model of spina bifida in which the *Zic2* gene is mutated. We identified two separate mechanisms that cause spina bifida. First, a developmental signalling pathway protein, bone morphogenetic protein (BMP), is overexpressed. This prevents the developing spine from rolling up and bending at so-called ‘dorsolateral hinge points’. However, this is not fully responsible for the spina bifida. The developing spinal tissue also has abnormal mechanical properties. This is due to excessive RhoA signalling and abnormal actomyosin accumulation in the neuroepithelium. This increases the stiffness of the developing spinal tissue and prevents it from rolling up into a tube and closing. We have therefore shown a dual mechanism for the spina bifida in *Zic2* mutant embryos.

**What are the potential implications of these results for your field of research?**

**S.R.-K.:** Open spina bifida is a complex disorder, caused by interactions between genetic and environmental factors. Children and adults with spina bifida require multiple surgical interventions and have a high degree of morbidity. Advances that prevent or reduce the severity of spina bifida are therefore of huge importance. Our work helps to characterise the signalling pathways downstream of *Zic2* that influence spinal neural tube closure. In particular, we have shown that upregulation of BMP and RhoA signalling are both implicated in the pathogenesis of spina bifida. This has connotations for primary prevention, early diagnosis and the development of novel therapies.

**S.E.:** We have identified a multi-pathway origin of spina bifida in the *Zic2^Ku^* mutant mouse. This suggests that genetic variants that lead to enhanced BMP and RhoA signalling may be candidates for the causation of human neural tube defects.

**What are the main advantages and drawbacks of the experimental system you have used as it relates to the disease you are investigating?**

**S.E./S.R.-K.:** The morphological mechanism of spinal neural tube closure in mice closely resembles that in humans. This is not the case in other model organisms, such as the frog, chick or zebrafish. A key strength of our experimental system was our whole-mouse embryo culture system. This allowed us to study neural tube closure *in vivo*. By adding signalling inhibitors to the embryo culture medium, we could directly study the effect of BMP and RhoA inhibition on spinal neural tube closure and morphogenesis.

The two main drawbacks of the system were cost and time. Mice are expensive to maintain and breed in the laboratory. It also takes a long time to gather and reproduce data from mouse embryos, particularly since we could not include heterozygous embryos in our experiments.

“[…] both overactivation and downregulation of RhoA signalling were associated with the same faulty actomyosin distribution in the neuroepithelium […]”

**Figure:**
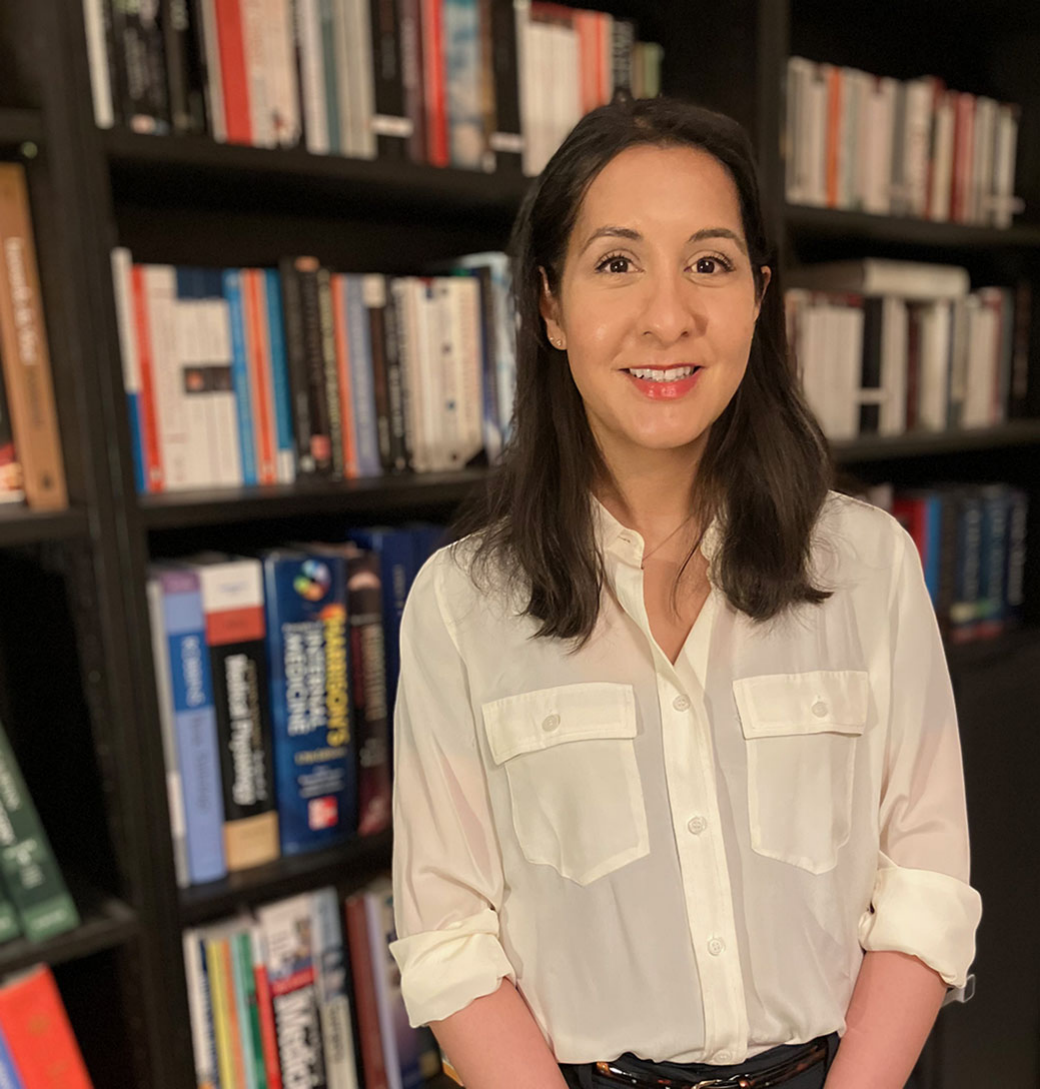
Saba Raza-Knight

**Figure:**
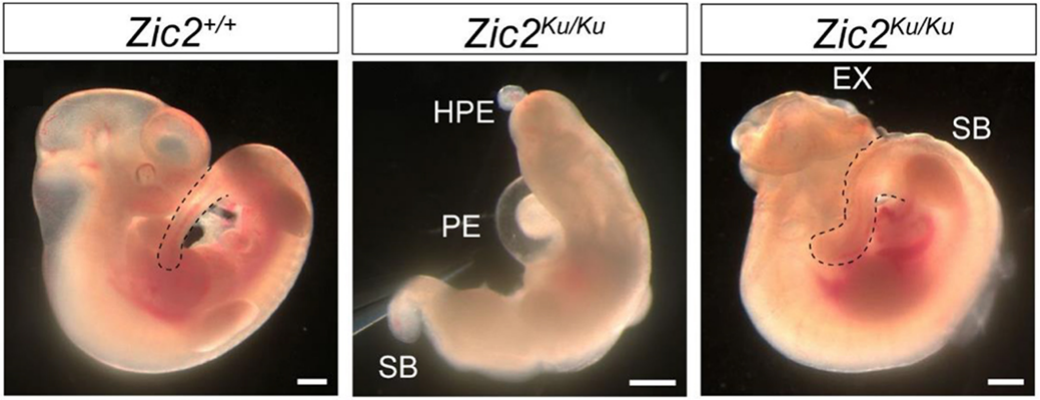
**Wild-type (left) and mutant (middle and right) *Zic2* mutant embryos, showing the range of phenotypes encountered.** These include the neural tube defects spina bifida (SB) and exencephaly (EX). The embryo in the middle has severe alobar holoprosencephaly (HPE) with a proboscis, and a pericardial effusion (PE). Dashed lines outline the posterior end of the embryo. Scale bars: 0.5 mm.

**What has surprised you the most while conducting your research?**

**S.E.:** Finding that the spinal neural tube could undergo zippering closure in the absence of typical dorsolateral hinge points was unexpected. I was also surprised that both overactivation and downregulation of RhoA signalling were associated with the same faulty actomyosin distribution in the neuroepithelium, showing that precise RhoA regulation is needed for normal spinal neural tube closure.

**S.R.-K.:** When we originally bred our *Zic2* mutant mice on a congenic C57BL/6 background, they developed additional severe developmental anomalies that precluded studying the spina bifida. We outcrossed them to another mouse strain (C3H/He) to improve their viability. I was surprised at the impact that the genetic background of the mouse had on phenotype. Another surprise was our inability to completely rescue the spina bifida phenotype based on the dual mechanism we identified. We could partially rescue the spina bifida in *Zic2^Ku^* mutants by culturing embryos with the BMP inhibitor dorsomorphin, or the myosin (a downstream effector of RhoA) inhibitor, Blebbistatin. However, culturing embryos with both of these inhibitors failed to completely rescue the spina bifida phenotype. This may be related to interactions between the two pathways, or synergistic toxicity of the two inhibitors.

**What do you think is the most significant challenge impacting your research at this time and how will this be addressed over the next 10 years?**

**S.E.:** Biological systems are dynamic entities, constantly submitting and adapting to constraints. Understanding how these systems operate in normal and diseased states is therefore a great challenge. I believe that future research in biology will have to be more multidisciplinary, including fields such as physics, chemistry, evolutionary biology, physiology and environmental science.

**S.R.-K.:** Neural tube defects are complex disorders, and I think that model organisms are necessary to study their pathogenesis. There is still much to be learned from mouse models. Validating and translating these findings to clinical practice will be the next challenge, and will require many steps. When prenatal surgery for spina bifida was being developed, larger mammalian models (sheep) were used before translation to humans. Collaboration between different disciplines will be essential.

**What changes do you think could improve the professional lives of scientists?**

**S.R.-K.:** Scientists may take career breaks for a number of reasons. In my case, these were related to medical training and maternity leave. Incentives to return to academia would encourage different career pathways and diversify the field. More financial support and job security for scientists would also improve the way we conduct research.

**What's next for you?**

**S.R.-K.:** I carried out this work as part of my MB PhD programme at University College London. Since leaving the lab, I have been completing my clinical training. I am now in the final few years of training as a neurosurgeon in Manchester, UK, where I am involved in clinical research. My current neurosurgical interests are in neurovascular disorders, cancer biology and genetics.

**S.E.:** In the current paper, we have shown the implication of BMP and RhoA signalling pathways for spinal neural tube closure. The next step will be to understand their exact involvement in the biomechanics of neurulation.
